# A CRITICAL REVIEW OF PAIN MANAGEMENT TRAINING PROGRAMS FOR FAMILY CAREGIVERS

**DOI:** 10.1093/geroni/igz038.2630

**Published:** 2019-11-08

**Authors:** Nai-Ching Chi, Emelia Barani, Ying-Kai Fu

**Affiliations:** 1 University of Iowa, Iowa city, Iowa, United States; 2 University of Washington, Seattle, Washington, United States

## Abstract

Many American family caregivers (FCs) manage hospice and palliative patients’ pain at home. However, FCs encounter many barriers to pain management due to inadequate training. Although some studies have designed pain management training programs for caregivers, the content and durations vary greatly. Thus, there is a lack of consistent recommendation on how to educate caregivers on pain management. The purpose of this study is to critically evaluate existing training programs to inform clinical practice and future program design. A literature review was conducted to search available articles published before June 2018 in databases including PubMed, CINAHL, PsycINFO, and Scopus. Search strategies used index and keyword methods. The inclusion criteria were peer-reviewed, research studies published in English that evaluated a pain management education or training for family caregivers. Twenty-seven studied were included. All studies improved either patients’ outcomes (e.g. pain intensity, hospital visits) or caregivers’ outcomes (e.g. knowledge, quality of life). Most studies (85%) had research teams provide caregivers with pain management education via multiple face-to-face training sessions and written booklet, while some studies (15%) had practicing nurses used videophones, web-based platforms, and telehealth to enhance the collaboration of care and pain management with caregivers. Providing adequate pain management training can improve patients’ and caregivers’ outcomes. However, in-person training programs are not practical for busy and overwhelmed caregivers. Researcher-delivered training sessions are not clinically adoptable. Future studies should develop pain management interventions that allow nurses to coach caregivers during routine visits and enhance their communication and collaboration with caregivers.

